# qRT-PCR Reference Gene Selection for the Discoloration of Tender Leaves in Hawk Tea (*Litsea coreana*)

**DOI:** 10.3390/cimb47020131

**Published:** 2025-02-18

**Authors:** Qianli Dai, Min Lu, Ximeng Yang, Chenggong Lei, Feiyi Huang, Xueping Hu, Xin Huang, Xiaolong Nie, Daojing Chen, Sicheng Huang, Hengxing Zhu

**Affiliations:** Chongqing Key Laboratory of Forest Ecological Restoration and Utilization in the Three Gorges Reservoir Area, Chongqing Academy of Forestry, Chongqing 400036, China; daiqianli126@126.com (Q.D.); yimi999@foxmail.com (M.L.); xm2020@email.swu.edu.cn (X.Y.); 18720973032@163.com (X.H.);

**Keywords:** *Litsea coreana*, hawk tea, real-time quantitative PCR, reference gene, tender leaves discoloration

## Abstract

To identify stable reference genes for qRT-PCR analysis across different developmental stages and color variations of tender leaves in *Litsea coreana*, seven candidate reference genes were selected based on existing transcriptome data. qRT-PCR was performed on tender leaves of *L. coreana* at various stages and under different color conditions. The stability of these genes was evaluated using GeNorm (version 2003), NormFinder (version 0953), BestKeeper (version 2003), and ReFinder software (version 2004). The most stable genes were selected, and the stability of the chosen reference genes was validated. *RPL* and *ACT* were the most stable genes across different leaf developmental stages, while *ACT* and *EF1-α* showed the highest stability across different leaf colors. Overall, *ACT* and *EF1-α* were the most stable reference genes for both developmental stages and color variations. *ACT* and *EF1-α* can be used as reliable reference genes for gene expression studies in the color change process of *L. coreana* tender leaves. This will provide a foundation for further research into the molecular mechanisms of leaf color changes and the development of color regulation genes in *L. coreana*.

## 1. Introduction

Plant leaves undergo color changes at various developmental stages and in response to different environmental conditions [1,2]. The primary cause of leaf discoloration is the relative variation in the content of chlorophyll, carotenoids, anthocyanins, and other compounds. These changes are influenced by both internal genetic factors and external environmental conditions, and are regulated by the microstructure, as well as the physiological and biochemical metabolic processes within leaf cells [1,3,4]. In recent years, significant advances have been made in the study of leaf color changes, from traditional physiological and biochemical approaches to high-throughput sequencing technologies in molecular biology and the identification of related functional genes. Central to these studies is the use of real-time quantitative PCR (qRT-PCR), a crucial method for gene expression analysis.

qRT-PCR is a widely used technique for assessing gene expression levels, combining conventional PCR with fluorescence detection to monitor amplification in real-time [5]. It offers advantages, such as low cost, high sensitivity, and strong specificity, making it essential in gene discovery and functional research [6,7]. However, certain factors, like sample quality, experimental conditions, RNA integrity, and reverse transcription efficiency, can impact qRT-PCR results [8], necessitating the use of appropriate reference genes for accurate normalization [9]. Typically, housekeeping genes involved in basic cellular functions or structural maintenance, such as actin (*Actin*), α/β-tubulin (*TUA*/*TUB*), 3-phosphoglycerate dehydrogenase (*GAPDH*), polyubiquitinase (*UBQ*), and 18S ribosomal RNA (*18SrRNA*), are selected as reference genes [5,8,9,10]. Recent studies, however, have shown that the expression stability of these genes can be affected by species, tissue type, and developmental stage [10,11].

*Litsea coreana* var. *lanuginosa*, an evergreen species within the Lauraceae family and belonging to the *Litsea* genus, is characterized by its young leaves that exhibit a range of colors, including red (purple) and green [12,13], which gradually mature to a green hue. This species, commonly known as leopard skin camphor, is widely distributed in Chongqing, with additional populations found in Sichuan and Guizhou [14,15]. The leaves of *L. coreana* are rich in bioactive compounds, such as flavonoids, polyphenols, polysaccharides, and coumarins, which are associated with beneficial effects, including blood sugar reduction, liver protection, and anti-inflammatory and antioxidant properties [16,17]. Due to these health-promoting properties, tea made from the leaves of this planet is highly valued among local populations. Overall, *L. coreana* is a woody plant with considerable potential for both ornamental and functional uses in food, medicine, and landscaping.

Despite the increasing interest in the genetic resources [14,15], whole genome analysis [14,15,18], and the development of functional components for metabolic tea drinks [19] derived from *L. coreana*, research on its molecular biology remains limited. Notably, there is a lack of studies focused on the screening and expression of reference genes in *L. coreana*. This study utilized real-time fluorescence quantitative PCR in combination with GeNorm, NormFinder, BestKeeper, and ReFinder software to identify suitable internal reference genes for investigating leaf color changes in the young leaves of *L. coreana* at various developmental stages and color variations. These findings provide a robust foundation for future molecular mechanism studies and the identification of key genes in the development of *L. coreana*.

## 2. Materials and Methods

### 2.1. Plant Materials

**Figure 1:**
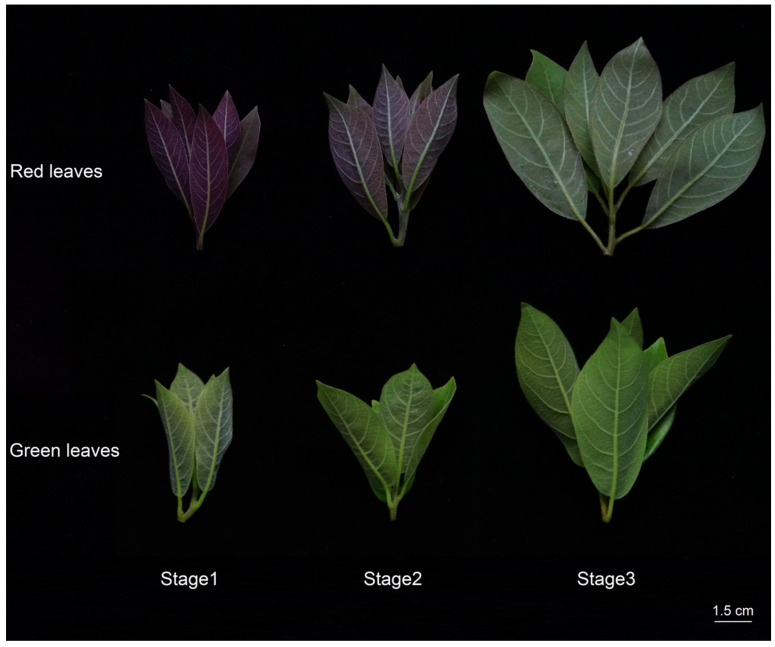
Phenotypic observations in *L. coreana* leaves. Morphological observations of red-leaved (R) and green-leaved (G) *L. coreana* at three developmental stages (S1–S3).

Fresh tender leaves with red (R) and green (G) coloration were collected from six 3-year-old, disease-free *L. coreana* trees grown under natural conditions in the nursery of the Chongqing Forestry Science Research Institute (20 °C, 80% relative humidity, 8 h photoperiod) between mid-March and early April 2022 (Figure 1). The leaves were classified into three developmental stages based on their color: red (S1), semi-red (S2), and green (S3). Immediately following collection, the leaves were frozen in liquid nitrogen and stored at −80 °C for subsequent analyses. The experiments were performed in triplicate.

### 2.2. Total RNA Extraction and cDNA Synthesis

RNA extraction was performed using the Hi Pure polysaccharide polyphenol plant total RNA extraction kit (TSP0202) from Beijing Tsingke Biotech Co., Ltd., Beijing, China. A SynScript^®^III RT SuperMix for qPCR (TSK314S) kit was used for reverse transcription and stored at −20 °C.

### 2.3. Selection of Candidate Reference Genes and Primer Synthesis

Based on a review of commonly used reference genes in plants, homologous genes were identified from the transcriptome database of *L. coreana* with leaf colors and developmental stages, constructed by the research institute. The 7 genes sequences (*Lc18S rRNA*, *LcUBC*, *LcACT*, *LcGAPDH*, *LcEF1-α*, *LcTUB*, and *LcRPL*) were then subjected to BLAST analysis on the NCBI database (https://blast.ncbi.nlm.nih.gov/Blast.cgi, accessed on 13 September 2024) to confirm the annotation and ensure the accuracy of the comparative results. The selected genes were used for the design of real-time quantitative PCR (qRT-PCR) primers, which were generated using the Primer Premier 5.0 software (Premier Biosoft International, Palo Alto, CA, USA). The primer details are provided in Table 1.

### 2.4. Quantitative PCR Program and Data Analysis

qRT-PCR was conducted using the AABI and QuantStudio StepOnePlus real-time fluorescence quantitative PCR instruments. The cDNA products obtained through reverse transcription were diluted threefold and used as templates for amplification with the ArtiCan^CEO^ SYBR qPCR Mix (Qingke, Beijing, China). The reaction mixture was prepared in a total volume of 20.0 μL, consisting of 10.0 μL ArtiCan^CEO^ SYBR qPCR Mix, 0.8 μL of each primer (10.0 μmol/L), 1.0 μL of cDNA template, and 7.4 μL of ddH_2_O. The PCR conditions included an initial denaturation at 95 °C for 5 min, followed by 40 amplification cycles (denaturation at 95 °C for 15 s, annealing at 60 °C for 20 s, and extension at 72 °C for 20 s, with fluorescence data collection at 72 °C). A final melting curve analysis was performed by heating from 65 °C to 95 °C, with continuous fluorescence collection at 0.1 °C intervals for verification of primer specificity.

The expression stability of the seven candidate internal reference genes at different developmental stages and leaf colors in *L. coreana* was assessed using four widely used algorithms: ΔCT [20], BestKeeper [21], GeNorm [22], and NormFinder [23]. Based on the results from these algorithms, the online tool RefFinder (https://blooge.cn/RefFinder/, accessed on 30 October 2024) was employed to perform a comprehensive ranking of the stability of the seven candidate genes. The genes were ranked from 1 to 7, with scores assigned accordingly (7 for the most stable gene and 1 for the least stable). The scores from each evaluation method were combined to provide a final stability ranking, enabling the selection of the most suitable internal reference genes for qRT-PCR analysis of the leaf color change process in *L. coreana*.

## 3. Results

### 3.1. Primer Specificity Analysis

**Figure 2:**
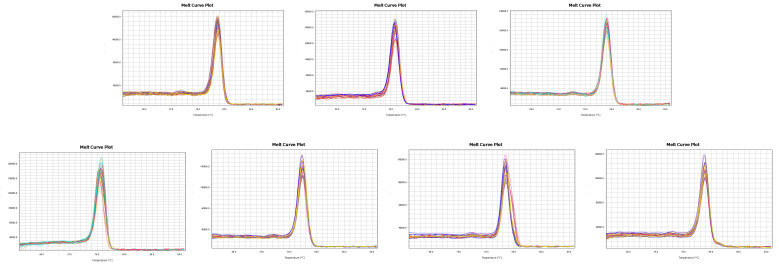
Melting curves with a single peak for the 7 candidate reference genes.

RT-qPCR verification of primer specificity revealed that the melting curves of the seven candidate reference genes exhibited single peaks (Figure 2), with no evidence of primer dimers or hairpin structures. The consistent amplification curves across replicate samples further confirmed that each reference gene exhibited optimal annealing temperatures and excellent primer specificity.

### 3.2. Candidate Reference Gene Expression Profile

**Figure 3:**
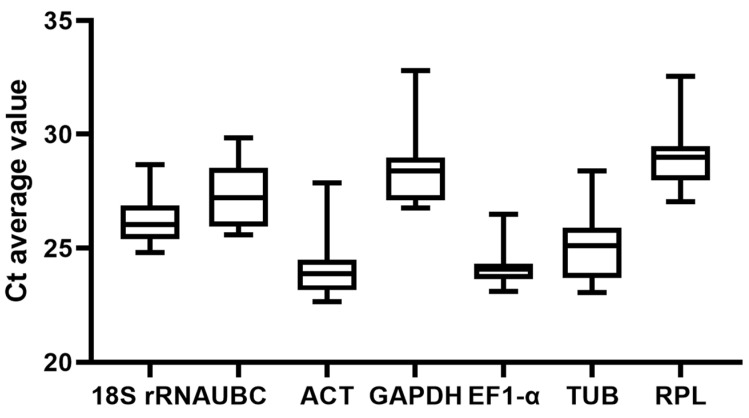
Distribution of Ct values of 7 candidate internal reference genes. The box line represents the range of concentrations of Ct values, the middle line of the box indicates the median, the top and bottom of the box indicate the distribution of the upper and lower quartiles, respectively, the top line of the box indicates the upper limit of the values, and the bottom line of the box indicates the lower limit of the values.

Gene expression levels are determined by the cycle threshold (Ct), which is inversely proportional to gene expression. As shown in Figure 3, the Ct values for the 7 candidate reference genes range from 22 to 33 cycles, indicating varying expression levels. Among these, *ACT* and *EF1-α* display relatively high and stable expression across different samples, with minimal variation in their Ct values. In contrast, *GAPDH* and *RPL* exhibit relatively lower expression levels. *UBC* and *TUB* show a wider expression range, with notable variations in their Ct values across different samples (Figure 3), suggesting significant differences in their expression.

### 3.3. Stability Evaluation of Candidate Reference Genes

Common algorithms for evaluating the stability of internal reference genes include ΔCT, BestKeeper, NormFinder, and GeNorm. The ΔCT method assesses stability based on the standard deviation of Ct values, where a lower standard deviation indicates greater stability. The BestKeeper algorithm evaluates stability by considering both the coefficient of variation and standard deviation of Ct values; a lower coefficient of variation and standard deviation indicate higher stability. Both NormFinder and GeNorm calculate an average expression stability index, which is inversely correlated with gene stability. These four algorithms were employed to assess the stability of seven candidate reference genes at different developmental stages and leaf color phases of *L. coreana*. Given the similarities and differences in the results across these software tools, ReFinder software was used for comprehensive analysis and performing geometric mean calculations on the stability rankings. A lower geometric mean indicates greater stability of the candidate internal reference genes.

#### 3.3.1. Analysis of ΔCT Method

As shown in Table 2, the analysis using the ΔCT algorithm reveals that *ACT* exhibits the highest stability in red tender leaves at different developmental stages. *RPL* is the most stable gene in green tender leaves across various stages, with *ACT* ranking second in this regard. Among leaves of different colors, *ACT* and *EF1-α* show the best stability. When considering both color and developmental stage, *ACT* is identified as the most stable reference gene. Although *UBC* demonstrates relatively stable expression in red tender leaves across stages, it is the least stable when analyzed across all stages. Additionally, *TUB* is identified as one of the most unstable candidate genes.

#### 3.3.2. NormFinder Analysis

NormFinder software works by calculating the value of the stability of each internal reference gene, meaning that the stable internal reference genes can be screened out. The judgment criterion is that the smaller the value, the better the stability of the internal reference gene, and vice versa, the worse the stability. Typically, NormFinder identifies a single optimal reference gene. In the case of *L. coreana* tree’s tender leaves at different stages, *ACT* and *RPL* are the most stable genes, while *TUB* is the least stable. Among the leaves of different colors, *EF1-α* and *ACT* show the most stable expression, whereas *UBC* displays poor stability. When combining different leaf colors and developmental stages, the *ACT* gene demonstrates the highest stability with a stability value of 0.100, while *UBC* is the most unstable with a value of 1.095. Overall, *ACT* ranks first or second in stability across all groupings (Table 3), whereas *TUB* consistently ranks lowest, indicating poor stability.

#### 3.3.3. BestKeeper Analysis

BestKeeper software identifies the optimal reference gene by analyzing the coefficient of variation (CV) and standard deviation (SD) of the Ct values. Smaller CV and SD values indicate greater stability [22]. When the SD exceeds 1, the reference gene is considered unstable. Similar to NormFinder, BestKeeper can only determine one optimal reference gene at a time. In the case of *L. coreana* tree’s tender leaves at various developmental stages and color phases, *EF1-α* and *18S rRNA* genes exhibit the highest stability, with SD values below 1 (Table 4). In contrast, the *GAPDH* gene shows the highest SD and CV values, indicating that it is the most unstable across most group analyses.

#### 3.3.4. GeNormGene Analysis

GeNorm software determines the most stable reference gene based on the average expression stability index (M), where a lower M value indicates greater stability. A threshold M value of 1.5 is used, with genes exceeding this value deemed unsuitable as internal reference genes. Additionally, GeNorm calculates the optimal number of reference genes required for normalization by analyzing paired variation values (V_n/n + 1_). If the V_n/n + 1_ value is below 0.15, the number of reference genes needed is n, while values above 0.15 indicate that an additional reference gene (n + 1) should be included [24].

**Figure 4:**
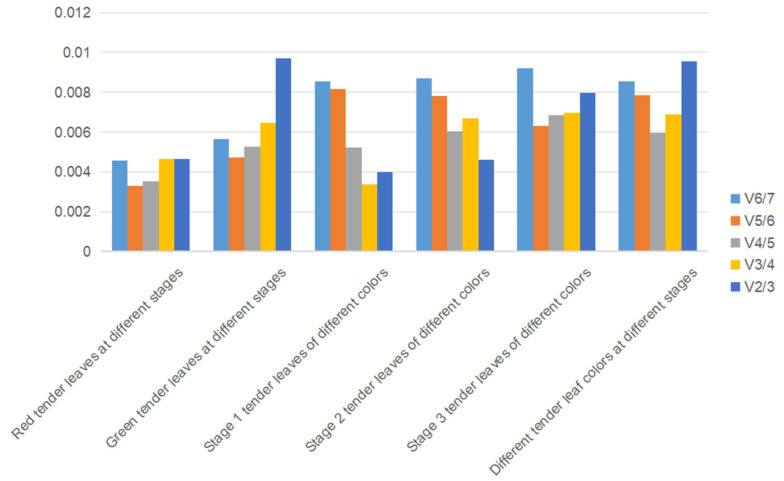
Paired variation (V_n/n +1_) analyzed by GeNorm software. GeNorm evaluates the ideal number of reference genes through paired variation analysis (Vn/n + 1). The threshold for Vn/n + 1 is set to 0.15, where n represents the optimal number of reference genes.

As shown in Table 5, the M values for all seven candidate genes in the stable and differently colored tender leaves of *L. coreana* tree at various developmental stages are below 1.5, indicating that all genes meet the criteria for reference gene screening using GeNorm software. In the red tender leaves at different stages, the *UBC* and *ACT* genes exhibit the highest stability, with a stability value of 0.142. For the green tender leaves, the *18S rRNA* and *RPL* genes display the most stable expression, with a stability value of 0.45. Under the different color stages of *L. coreana* tree, *ACT* and *EF1-α*, as well as *GAPDH* and *ACT*, show the most stable combinations, with stability values of 0.187 and 0.177–0.355, respectively. Based on the analyses of different colors and stages, the genes *ACT* and *GAPDH* are identified as the most stable, with a stability value of 0.397 and a V_2/3_ value below 0.15, indicating that the selection of two internal reference genes meets the requirements for gene normalization (Figure 4).

#### 3.3.5. RefFinder Analysis

Based on the comprehensive analysis conducted using ReFinder software (Table 6), the *ACT* and *UBC* genes exhibit the highest stability in red tender leaves across different developmental stages, making them suitable as internal reference genes for this stage. For green tender leaves at various stages, *RPL* and *18S rRNA* genes rank first and second in stability, respectively, and are recommended as internal reference genes for green leaves. In the analysis of tender leaves of different colors and stages, *ACT* and *EF1-α* are identified as the most stable genes.

The stability of candidate genes varies across different stages and leaf colors. After ranking and scoring the stability of the candidate genes for each stage of *L. coreana* tree’s tender leaves, and summing the results (Table 7), *RPL* and *ACT* were found to be the most stable internal reference genes. Overall, considering all analysis results, *ACT* and *EF1-α* emerged as the most stable genes, while *TUB* and *UBC* were ranked lower and are, therefore, unsuitable for use as internal reference genes in studying the color change process of young leaves in the leopard skin camphor tree.

### 3.4. Identification of Reference Genes Through Screening

**Figure 5:**
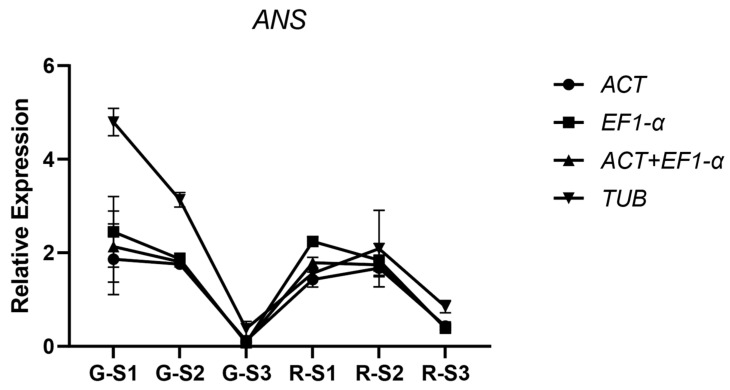
Verifying the stability of *L. coreana* reference genes using ANS. We use RT-qPCR to evaluate the expression pattern of *ANS* in leaves at different leaf color stages, and normalize gene expression data using *LcEF1-α*, *LcACT*, or a combination of both. We analyze gene expression using the 2^−∆Ct^ method. G-S3 (green leaves—stage 3); G-S2 (green leaves—stage 2); G-S1 (green leaves—Stage 1); R-S3 (red leaves—stage 3); R-S2 (red leaves—stage 2); R-S1 (red leaves—stage 1).

To validate the selected reference genes, qRT-PCR was employed to measure the expression of *ANS* genes in the tender leaves of *L. coreana* at different stages and colors (Figure 5). The expression of the *ANS* genes was normalized using the most stable reference gene combination and compared to the least stable reference gene, *TUB.* When the gene expression levels were standardized using the stable reference genes, the expression patterns displayed similar trends. In contrast, normalization with *TUB* revealed notable discrepancies compared to other groups, indicating that the selected stable reference genes are reliable for use in different leaf colors and stages of the camphor tree.

## 4. Discussion

Gene expression analysis is a critical area of research in molecular biology, with qRT-PCR being the most widely used technique for assessing gene expression levels. It is commonly employed to investigate gene expression patterns across various samples, tissues, developmental stages, and under specific experimental conditions [25]. Accurate selection of reference genes is essential for the normalization of gene expression data during such analyses [26,27]. Although housekeeping genes have traditionally been used as internal reference genes, recent studies have revealed that reference genes lack universality and must be selected based on the specific species or experimental conditions [28,29]. For instance, *GAPDH* is consistently stable in certain species, such as *Paeonia veitchii* [30], but it is considered an unsuitable reference gene in other species, like *Luffa cylindrica* and *Boehmeria nivea* [31,32]. Similarly, the *18S rRNA* gene exhibits stable expression under various treatments in *Taxus chinensis* [33], yet shows significant variability in its expression across different treatments in *Isatis indigotica* and *Avena sativa* affected by abiotic stresses [34,35]. Currently, no reference gene has been identified that is universally stable across all experimental conditions. Therefore, researchers must identify appropriate reference genes tailored to their specific experimental setups for accurate quantitative analysis.

In the selection of internal reference genes for real-time quantitative PCR (qRT-PCR) across various developmental stages and leaf colors in plants, GeNorm, NormFinder, BestKeeper, and ΔCT are the four most widely utilized methods. The recent literature indicates that GeNorm is the most frequently employed method, followed by NormFinder, while BestKeeper and ΔCT are typically applied in specific contexts or smaller-scale studies. GeNorm has emerged as the predominant tool due to its capacity to simultaneously assess the stability of multiple genes and its robust performance under complex experimental conditions. For instance, Vandesompele et al. first introduced GeNorm, showcasing its broad applicability across diverse plant species and tissues [22]. NormFinder is the second most frequently used method, owing to its precision in ranking the stability of multiple genes, particularly in studies involving multiple experimental conditions. Developed by Andersen et al., NormFinder has been widely adopted in various research contexts [23]. BestKeeper and ΔCT, while also commonly used, are often employed in more limited or specific scenarios. BestKeeper assesses gene stability by calculating the coefficient of variation and standard deviation of Ct values, though it is noted for its sensitivity to extreme values [21]. The ΔCT method, valued for its simplicity and directness, is frequently utilized for rapid evaluations, albeit with the limitation of being unable to assess multiple genes simultaneously [36,37]. In this research, four analytical methods and one online tool were employed to assess the stability of seven candidate reference genes. The results revealed that while all seven reference genes exhibited stable expression in the leaves of *L. coreana*, their stability rankings varied slightly. According to the ΔCT and NormFinder methods, *ACT* and *EF1-α* were identified as the most stable genes. In contrast, GeNorm ranked *ACT* first and *EF1-α* fourth. Similarly, in BestKeeper, *EF1-α* was ranked first and *ACT* third. These discrepancies in rankings can be attributed to the different statistical algorithms used in each analysis method.

Given the limitations of each individual software, a comprehensive analysis was conducted using RefFinder to integrate the results from the four tools. The stability ranking of the seven candidate reference genes in the leaves of *L. coreana* was determined as follows: *ACT* > *EF1-α* > *18SrRNA* > *RPL* > *GAPDH* > *UBC* > *TUB*. Based on the paired variation values (V_n/(n + 1)_) analyzed by GeNorm, where the ratio was found to be less than 0.15, the study recommends using two reference genes in combination to ensure greater accuracy in gene normalization, considering the experimental costs and sample size. Previous studies have also suggested that employing multiple reference genes is a more effective approach for correcting systematic biases in quantitative analyses [38].

Anthocyanins play a crucial role in determining leaf color. In the transcriptome data from our research group, we identified differential expression of *ANS* during the color change process of the tender leaves of *L. coreana*, which supports the potential utility of the selected reference genes. The internal reference genes with the highest stability, as determined by software analysis, were *ACT* and *EF1-α*. These two genes, alone or in combination, were used to normalize the expression level of the *ANS* gene in *L. coreana* leaves. *ACT* and *EF1-α* are widely recognized as stable housekeeping genes in eukaryotic organisms and have been previously reported as ideal reference genes in various ornamental tree species with different leaf colors, such as *Lagerstroemia indica* and *Schima superba* [39,40]. Furthermore, while *UBC* and *TUB* exhibited poor stability in the leaves of *L. coreana* in this study, they have shown good stability in other species, such as *Oryza sativa* and *Chenopodium quinoa* [41,42]. This highlights the species-specific nature of reference gene stability and underscores the lack of universality in internal reference gene selection.

## 5. Conclusions

This study identified seven candidate reference genes (*ACT*, *EF1-α*, *18SrRNA*, *RPL*, *GAPDH*, *UBC*, and *TUB*) based on prior research and transcriptome data from our research group. The stability of each candidate gene was assessed using qRT-PCR and various internal reference gene evaluation methods, including ΔCT, GeNorm, NormFinder, and BestKeeper. The results indicated that *ACT* and *EF1-α* were the most stable and suitable reference genes for different developmental stages and leaf colors of *L. coreana*. Additionally, the expression of the anthocyanin biosynthesis gene *ANS* was used to further validate the reliability of the software-based stability analysis. This study enhances the selection process for internal reference genes in the Lauraceae family, providing a theoretical foundation for investigating the molecular mechanisms underlying leaf color formation in *L. coreana*. Furthermore, it offers valuable insights for selecting appropriate reference genes in other plant species, benefiting both our research group and the broader scientific community.

## Figures and Tables

**Table 1:** Primer design for internal reference genes and *ANS* genes.

Gene Name	Primer Name	Primer (Forward/Reverse)	Size/bp
18S rRNA gene (*18S rRNA*)	nc1-18S rRNA-F	CGTCACTGCAACTCTCTCCA	130
	nc1-18S rRNA-R	GGCGTGGTTGTTGACTTTCC	
Ubiquitin-conjugating C (*UBC*)	nc2-UBC-F3	TCACTGGAAAGACCATAACGCT	108
	nc2-UBC-R3	TTAGACGTTGCTGATCCGGG	
Actin (*ACT*)	nc3-ACT-F	CCCATCCCGACCATTACACC	106
	nc3-ACT-R	GAACTGGGATGGTCAAGGCA	
Glyceraldehyde-3-phosphate dehydrogenase (*GAPDH*)	nc4-GAPDH-F	GGGATCTCTGATGGGTCCCT	188
	nc4-GAPDH-R	AGGGATGATGTTGAGGTGGT	
Elongation Factor1-alpha (*EF1-α*)	nc5-EF1-α-F	TGATCCAGCAAAGGAGGCAG	107
	nc5-EF1-α-R	GGGAGGTGTGGCAATCAAGA	
Tubulin beta (*TUB*)	nc6-TUB-F	TGGCAACTCCACCTCAATCC	187
	nc6-TUB-R	CGCTGTTGCATCCTGGTACT	
Ribosomal protein l (*RPL*)	nc7-RPL-F	GCAGCGGACAGAAAGAAGGA	114
	nc7-RPL-R	CTTTCCTGTGCTGGAGCTGA	
Anthocyanidin synthase (*ANS*)	nc18-ANS-1(Mei)-F	GCCCTCACCTTCATCCTCAC	126
	nc18-ANS-1(Mei)-R	GAGGATCTCAAGGCTGTCGC	

**Table 2:** ΔCT software (version 2003) analysis results.

Rank	Stability of Tender Leaves at Different Stages				Stability of Tender Leaves of Different Colors						Stability of Different Tender Leaf Colors at Different Stages	
	Red Tender Leaves at Different Stages		Green Tender Leaves at Different Stages		Stage 1 Tender Leaves of Different Colors		Stage 2 Tender Leaves of Different Colors		Stage 3 Tender Leaves of Different Colors			
	Gene	Stability Values	Gene	Stability Values	Gene	Stability Values	Gene	Stability Values	Gene	Stability Values	Gene	Stability Values
1	*ACT*	0.35	*RPL*	0.54	*EF1-α*	0.48	*ACT*	0.57	*EF1-α*	0.66	*ACT*	0.66
2	*UBC*	0.37	*ACT*	0.58	*ACT*	0.49	*EF1-α*	0.57	*ACT*	0.74	*RPL*	0.74
3	*RPL*	0.38	*18SrRNA*	0.62	*GAPDH*	0.51	*GAPDH*	0.62	*RPL*	0.76	*EF1-α*	0.75
4	*18SrRNA*	0.39	*EF1-α*	0.68	*RPL*	0.56	*18SrRNA*	0.7	*18SrRNA*	0.81	*18SrRNA*	0.75
5	*GAPDH*	0.48	*UBC*	0.73	*18SrRNA*	0.62	*RPL*	0.77	*GAPDH*	0.82	*GAPDH*	0.77
6	*EF1-α*	0.5	*GAPDH*	0.75	*TUB*	1.02	*TUB*	1.04	*TUB*	0.91	*TUB*	1.02
7	*TUB*	0.59	*TUB*	0.8	*UBC*	1.05	*UBC*	1.18	*UBC*	1.27	*UBC*	1.17

**Table 3:** NormFinder software analysis results.

Rank	Stability of Tender Leaves at Different Stages				Stability of Tender Leaves of Different colors						Stability of Different Tender Leaf Colors at Different Stages	
	Red Tender Leaves at Different Stages		Green Tender Leaves at Different Stages		Stage 1 Tender Leaves of Different Colors		Stage 2 Tender Leaves of Different Colors		Stage 3 Tender Leaves of Different Colors			
	Gene	Stability Values	Gene	Stability Values	Gene	Stability Values	Gene	Stability Values	Gene	Stability Values	Gene	Stability Values
1	*ACT*	0.155	*RPL*	0.174	*EF1-α*	0.093	*ACT*	0.088	*EF1-α*	0.175	*ACT*	0.1
2	*RPL*	0.183	*ACT*	0.287	*ACT*	0.093	*EF1-α*	0.136	*ACT*	0.359	*18SrRNA*	0.395
3	*UBC*	0.201	*18SrRNA*	0.376	*GAPDH*	0.107	*GAPDH*	0.21	*18SrRNA*	0.502	*EF1-α*	0.397
4	*18SrRNA*	0.206	*EF1-α*	0.493	*RPL*	0.252	*18SrRNA*	0.349	*RPL*	0.513	*RPL*	0.452
5	*GAPDH*	0.39	*UBC*	0.596	*18SrRNA*	0.373	*RPL*	0.561	*GAPDH*	0.579	*GAPDH*	0.478
6	*EF1-α*	0.414	*GAPDH*	0.604	*TUB*	0.988	*TUB*	0.972	*TUB*	0.762	*TUB*	0.919
7	*TUB*	0.533	*TUB*	0.687	*UBC*	1.016	*UBC*	1.134	*UBC*	1.205	*UBC*	1.095

**Table 4:** BestKeeper software analysis results.

Rank	Stability of Tender Leaves at Different Stages						Stability of Tender Leaves of Different Colors									Stability of Different Tender Leaf Colors at Different Stages		
	Red Tender Leaves at Different Stages			Green Tender Leaves at Different Stages			Stage 1 Tender Leaves of Different Colors			Stage 2 Tender Leaves of Different Colors			Stage 3 Tender Leaves of Different Colors					
	Gene	SD	CV	Gene	SD	CV	Gene	SD	CV	Gene	SD	CV	Gene	SD	CV	Gene	SD	CV
1	*EF1-α*	0.5	2.09	*EF1-α*	0.64	2.63	*EF1-α*	0.13	0.56	*EF1-α*	0.54	2.24	*18SrRNA*	0.66	2.44	*EF1-α*	0.56	2.32
2	*18SrRNA*	0.72	2.74	*18SrRNA*	0.95	3.64	*GAPDH*	0.15	0.55	*18SrRNA*	0.65	2.47	*EF1-α*	0.84	3.39	*18SrRNA*	0.83	3.19
3	*RPL*	0.8	2.83	*RPL*	1.02	3.43	*ACT*	0.18	0.79	*TUB*	0.7	2.83	*RPL*	1.12	3.74	*ACT*	0.92	3.81
4	*UBC*	0.81	2.91	*ACT*	1.08	4.43	*18SrRNA*	0.23	0.91	*ACT*	0.73	3.02	*UBC*	1.13	4.03	*RPL*	1	3.44
5	*ACT*	0.86	3.6	*TUB*	1.09	4.21	*RPL*	0.34	1.2	*GAPDH*	0.77	2.71	*TUB*	1.15	4.36	*TUB*	1.1	4.37
6	*TUB*	0.94	3.87	*UBC*	1.22	4.57	*UBC*	0.76	2.87	*RPL*	0.86	3	*ACT*	1.31	5.22	*UBC*	1.12	4.09
7	*GAPDH*	1.05	3.75	*GAPDH*	1.31	4.54	*TUB*	0.84	3.49	*UBC*	1.06	3.86	*GAPDH*	1.41	4.74	*GAPDH*	1.17	4.11

**Table 5:** GenormGene software analysis results.

Rank	Stability of Tender Leaves at Different Stages				Stability of Tender Leaves of Different Colors						Stability of Different Tender Leaf Colors at Different Stages	
	Red Tender Leaves at Different Stages		Green Tender Leaves at Different Stages		Stage 1 Tender Leaves of Different Colors		Stage 2 Tender Leaves of Different Colors		Stage 3 Tender Leaves of Different Colors			
	Gene	Stability Values	Gene	Stability Values	Gene	Stability Values	Gene	Stability Values	Gene	Stability Values	Gene	Stability Values
1	*UBC*	0.142	*18SrRNA*	0.45	*ACT*	0.187	*GAPDH*	0.177	*GAPDH*	0.355	*ACT*	0.397
2	*ACT*	0.142	*RPL*	0.45	*EF1-α*	0.187	*ACT*	0.177	*ACT*	0.355	*GAPDH*	0.397
3	*RPL*	0.244	*EF1-α*	0.503	*GAPDH*	0.215	*EF1-α*	0.269	*RPL*	0.521	*RPL*	0.502
4	*18SrRNA*	0.291	*ACT*	0.536	*RPL*	0.262	*18SrRNA*	0.378	*EF1-α*	0.58	*EF1-α*	0.588
5	*EF1-α*	0.337	*UBC*	0.569	*18SrRNA*	0.349	*RPL*	0.485	*TUB*	0.626	*18SrRNA*	0.618
6	*GAPDH*	0.377	*GAPDH*	0.62	*TUB*	0.525	*TUB*	0.616	*18SrRNA*	0.686	*TUB*	0.704
7	*TUB*	0.438	*TUB*	0.67	*UBC*	0.674	*UBC*	0.776	*UBC*	0.854	*UBC*	0.837

**Table 6:** RefFinder software analysis results.

Rank	Stability of Tender Leaves at Different Stages				Stability of Tender Leaves of Different Colors						Stability of Different Tender Leaf Colors at Different Stages		Evaluation
	Red Tender Leaves at Different Stages		Green Tender Leaves at Different Stages		Stage 1 Tender Leaves of Different Colors		Stage 2 Tender Leaves of Different Colors		Stage 3 Tender Leaves of Different Colors				
	Gene	Stability Values	Gene	Stability Values	Gene	Stability Values	Gene	Stability Values	Gene	Stability Values	Gene	Stability Values	
1	*ACT*	1.5	*RPL*	1.32	*EF1-α*	1	*ACT*	1.41	*EF1-α*	1.68	*ACT*	1.32	7
2	*UBC*	2.21	*18SrRNA*	2.06	*ACT*	1.86	*EF1-α*	1.86	*ACT*	2.21	*EF1-α*	2.45	6
3	*RPL*	2.71	*EF1-α*	2.63	*GAPDH*	2.71	*GAPDH*	2.59	*18SrRNA*	2.91	*18SrRNA*	2.99	5
4	*18SrRNA*	3.36	*ACT*	2.83	*RPL*	4.23	*18SrRNA*	3.36	*RPL*	3.22	*RPL*	3.13	4
5	*EF1-α*	3.66	*UBC*	5.23	*18SrRNA*	4.73	*TUB*	5.05	*GAPDH*	3.64	*GAPDH*	3.64	3
6	*GAPDH*	5.69	*GAPDH*	6.24	*TUB*	6.24	*RPL*	5.23	*TUB*	5.48	*TUB*	5.73	2
7	*TUB*	6.74	*TUB*	6.44	*UBC*	6.74	*UBC*	7	*UBC*	6.09	*UBC*	6.74	1

**Table 7:** Ranking of candidate gene stability scores.

Rank	Gene	Value
1	*ACT*	37
2	*EF1-α*	34
3	*18SrRNA*	27
4	*RPL*	26
5	*GAPDH*	20
6	*UBC*	13
7	*TUB*	11

## Data Availability

Data are contained within the article.
